# Predisposition of the Common *MC4R* rs17782313 Female Carriers to Elevated Obesity and Interaction with Eating Habits

**DOI:** 10.3390/genes14111996

**Published:** 2023-10-25

**Authors:** Danyel Chermon, Ruth Birk

**Affiliations:** Nutrition Department, Health Sciences Faculty, Ariel University, Ariel 40700, Israel; danyel.chermon@msmail.ariel.ac.il

**Keywords:** *MC4R*, obesity, single nucleotide polymorphisms, eating behavior

## Abstract

The global rise in obesity is attributed to genetic predisposition interaction with an obesogenic environment. Melanocortin 4 receptor (*MC4R*) rs17782313 polymorphism has been linked to common obesity with varying influence across different populations. *MC4R* is a crucial player in the leptin proopiomelanocortin pathway that regulates weight hemostasis. We aimed to study *MC4R* rs17782313 and its interaction with eating behaviors on obesity predisposition in the Israeli population. Adults’ (*n* = 5785, >18 y) genotype and anthropometric and demographic data were analyzed using logistic regression models adjusting for age, sex, T1DM, and T2DM. *MC4R* rs17782313 significantly predisposes to elevated obesity risk under the recessive and additive models (OR = 1.38, 95% CI: 1.1–1.72, *p* = 0.005 and OR = 1.1, 95% CI: 1.01–1.2, *p* = 0.03, respectively) adjusted for confounders (age, sex, T1DM, and T2DM). Stratification by sex demonstrated that carrying the common *MC4R* rs17782313 is significantly associated with an elevated predisposition to obesity under the recessive model among females only (OR = 1.41, 95% CI: 1.09–1.82, *p* = 0.01), with an average of 0.85 BMI increment compared with wild type and one risk allele carriers. *MC4R* rs17782313 significantly interacted with several eating behaviors to enhance the risk of obesity. Our findings demonstrate that *MC4R* rs17782313 homozygous female carriers are significantly predisposed to obesity amplified by eating behaviors.

## 1. Introduction

Obesity is a complex and multifaceted health condition characterized by an excessive accumulation of body fat; it is often measured using the Body Mass Index (BMI). It has evolved into a global epidemic that grows continuously, affecting both developed and developing nations [1]. The etiology of obesity is multifactorial, involving a combination of genetic, environmental, and behavioral factors. Genetic predisposition plays a role, as evidenced by twin and family studies with an estimated 40–75% heritability for obesity [2,3]. However, the rapid rise in obesity rates cannot be solely attributed to genetic factors and suggests a significant influence of environmental and lifestyle changes [4]. The widespread epidemic of common obesity is significantly exacerbated by an environment conducive to weight gain; it is marked by factors such as high-calorie foods, a lack of physical activity, environmental pollutants, rapid consumption of meals, oversized food portions, sugar-sweetened beverages, sedentary behavior related to screen time, inadequate sleep, and excessive consumption of simple carbohydrates and sugars [4,5]. Moreover, the prevalence of obesity varies significantly among different age groups, sex, and ethnicities, indicating that the risk factors for obesity are not uniformly distributed across populations [6]. The BMI is the prevalent, most used index for obesity classification due to its simplicity. BMI is calculated by dividing body weight in kilograms by height in meters squared. According to the BMI, individuals are allocated to five different categories as follows: 18.5–24.9 kg/m^2^: normal range, 25.0–29.9 kg/m^2^: overweight, 30.0–34.9 kg/m^2^: class 1-obesity, 35.0–39.9 kg/m^2^: class 2-obesity, equal or greater than 40 kg/m^2^: class 3-obesity [7]. Obesity is a well-established risk factor for a myriad of chronic medical and psychological conditions, including cardiovascular diseases, type 2 diabetes, certain types of cancer, depression, and reduced quality of life [8,9]. The economic burden of obesity is also substantial, with increased healthcare costs and lost productivity affecting both individuals and society [10]. Beyond healthcare costs, obesity significantly impacts workforce productivity and disability rates [11]. Eating behaviors have been increasingly recognized as a critical factor in the development and maintenance of obesity. Behaviors such as emotional eating, binge eating, overeating, and night-time eating have been shown to contribute to weight gain and obesity [12,13]. These behaviors often serve as coping mechanisms for stress, emotional disturbances, or even boredom, leading to the consumption of high-calorie, nutrient-poor foods [14].

Common polygenic obesity is attributed to a range of several to hundreds of genetic polymorphisms, each with a relatively small effect, predisposing the carrier to develop obesity, particularly with exposure to an obesogenic environment [14]. Genetic alternations to genes involved in the central nervous system (CNS) and neuronal pathways that control the hedonic aspects of food intake have emerged as the major drivers of elevated body weight for both monogenic and polygenic obesity [15,16]. The interaction between genetic predisposition and environmental factors referred to as “gene-environment interaction”, can significantly modulate the risk of obesity [17].

**Figure 1:**
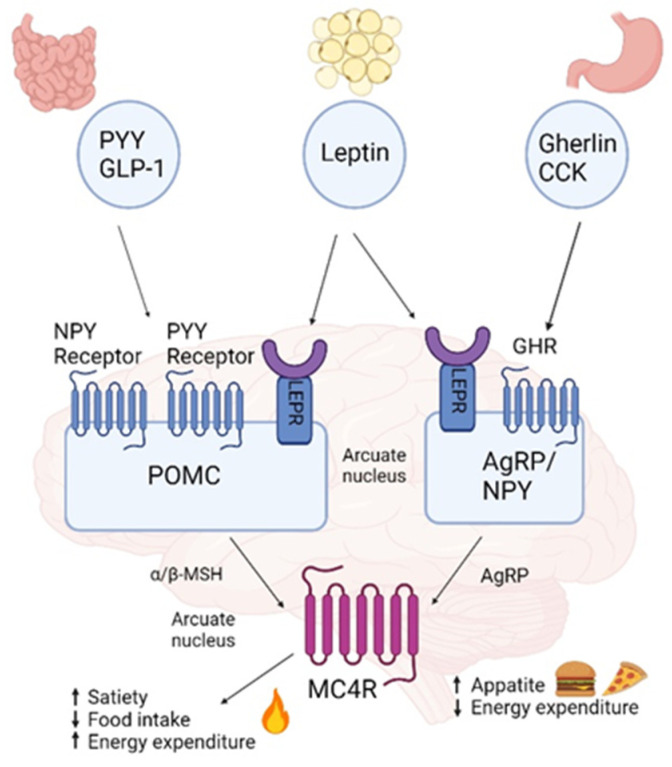
Leptin melanocortin pathway. Leptin is secreted by adipose tissue and activates leptin receptors (LEPR) in the arcuate nucleus: proopiomelanocortin (POMC) expressing neurons are activated accelerating α-melanocyte stimulating hormone (α-MSH), which activates the melanocortin-4 receptor (*MC4R*) allocated in the paraventricular nucleus (PVN), leading to reduced food intake and increased energy expenditure whilst inhibiting the secretion of the *MC4R* inverse agonist agouty related protein (AgRP) by AgRP/NPY [22,24,25,45]. Created with BioRender.com. (accessed on 27 September 2023).

The hypothalamus plays a pivotal role in integrating the regulation of food intake by sensing circulating levels of metabolites and altering eating patterns based on the concentrations of these molecules [17]. It is a key player in the neural leptin-melanocortin pathway, which includes melanocortin 4 receptor (*MC4R*) and is involved in body weight homeostasis and food intake, with genetic disruption resulting in extreme obesity and more subtle polymorphic variations influencing the population distribution of body weight [18]. *MC4R* is a G protein-coupled receptor that belongs to the largest family of transmembrane receptors in humans, the melanocortins, consisting of nearly 800 distinct genes and their corresponding gene products [19]. Located in chromosome 18q21.3., *MC4R* is a major player in the leptin–melanocortin pathway and has an essential role in food intake and energy homeostasis (Figure 1). Distributed widely throughout the central nervous system, *MC4R* is stimulated by the binding of α-melanocyte-stimulating hormone (α-MSH) released from proopiomelanocortin (POMC) neurons, resulting in the exocytosis of brain anorexigenic signals that regulate the satiety signal [20,21]. In the pathway, in brief, leptin released to the bloodstream by adipocytes in response to over-size and nutrient over-flow crosses the blood-brain barrier and binds to the leptin receptor on the surface of POMC neurons. Leptin binding to its receptor results in the secretion of α-MSH, which acts on *MC4R* neurons to increase energy expenditure and decrease energy intake [22]. In parallel, leptin binds to the leptin receptor on the surface of neurons, localized in the arcuate nucleus (ARC), resulting in Agouti-related peptide (AgRP) and neuropeptide Y (NPY) expression and the release of MC4R inhibition [23]. Other anorexigenic chemoreceptors and hormone players released by the gut, such as cholecystokinin (CCK) and glucagon-like peptide (GLP)-1, also bind to their respective receptors, stimulate POMC neurons, and contribute to reducing appetite and increased energy expenditure. Peptide YY (PYY) binds to its receptors on NPY/AgRP neurons to inhibit NPY/AgRP signaling. Orexigenic players such as Gherlin secreted from the stomach, also regarded as the “hunger hormone”, stimulate NPY/AgRP neurons to promote appetite and prevent satiety signals [22,24,25]. Deleterious mutations truncate or alter protein function in any of the genes along the leptin-melanocortin pathway, including *MC4R*, often causing early onset and severe monogenic obesity [26]. More than 200 *MC4R* variants have been identified over the past two decades, inherited primarily in an autosomal dominant pattern, with obesity resulting from only one affected allele mutation [27,28]. However, *MC4R* variants of homozygous and mixed inheritance patterns have also been identified in consanguineous families and linked with severe obesity [29,30]. To date, *MC4R* genetic variation is the most common (2–8% of common obesity) cause of early-onset and the most severe monogenic non-syndromic obesity known, influencing eating behavior and hyperphagia [19,26,31]. *MC4R* expression is affected differently by each mutation, and the obesity phenotype is determined by variable penetrance, expressivity, and allelic heterogeneity that contributes to different pathogenic mechanisms. Additionally, *MC4R* signaling is coupled to the three main heterotrimeric G proteins: Gs (stimuli), Gi (inhibition), and Gq. Thus, depending on the genetic variation, both loss-of-function and gain-of-function were identified in the population. Consequently, the obesity phenotype resulting from *MC4R* mutation can range from lean to morbid obesity [32]. Most known *MC4R* gene variants result in loss-of-function. However, about 15% of them result in gain-of-function, which protects against obesity and is associated with a favorable metabolic profile; these include, for instance, I251L and V103I [28,33]. *MC4R* variants that do not completely disrupt protein function may lead to influence the individual’s polygenic susceptibility to obesity [34,35]. Moreover, *MC4R* gene variation can interact with other obesity-linked genes and thereby elevate obesity risk. For example, common variants near *MC4R* and FTO seem to have additive effects on BMI [36].

The near *MC4R* rs17782313 single nucleotide polymorphism (SNP), located 188 kb downstream of the *MC4R* gene, has been associated with BMI and weight regulation in early life [36,37]. Notably, the rs17782313 C-allele has also been linked to BMI during childhood and adolescence [38]. Additionally, although scarcely studied, the rs17782313-C variant has been linked to eating behavior traits [39]. Several studies have shown that *MC4R* rs17782313 SNP carriers have an increased risk for obesity among different populations, though with varying influence across different populations [40]. Moreover, the *MC4R* rs17782313 variant has been implicated in metabolic pathways that influence energy balance, affecting weight regulation [41]. *MC4R* rs17782313 variant has also been associated with other metabolic processes that could indirectly contribute to obesity. For instance, this variant has been associated with altered lipid metabolism and obesity-related cardio-metabolic traits [42,43], which could further exacerbate obesity risk. Furthermore, the *MC4R* rs17782313 has been studied with other obesity-related co-morbidities such as type 2 diabetes and cardiovascular diseases [44]. This further emphasizes this genetic variant’s multi-faceted impact on health outcomes and obesity-related co-morbidities. Lately, research on obesity-linked variants from different populations carrying different genetic architectures has been investigated, disclosing the importance of addressing genetic risk in different populations to better calculate genetic risk. We aimed to study the association of *MC4R* rs177823313 with obesity risk and eating habits in the Israeli population. Given the complex interplay of genetic and environmental factors, as well as the role of the hypothalamus and *MC4R* in obesity, an intricate approach is essential for understanding and treating this condition.

## 2. Materials and Methods

### 2.1. Participants

Adults (≥ 18 years, *n* = 5785), out of which 69.5% were females, were included in the analysis. The research data source was from the Israeli registry database of Lev Hai Genetics LTD–MyGenes (Registry #700068969). Anonymous genetic data were employed for the analysis. Ethical approval for the study was granted by Ariel University’s ethical committee (Approval #AU-HEA-RB-20220214). The study sample excluded individuals younger than 18 years of age, those with genetic diseases, or those with incomplete anthropometric data.

### 2.2. Anthropometric and Genetic Data

Self-reported metrics were used for weight and height, with height indicated in centimeters and weight in kilograms. BMI was determined using the formula weight/(height)^2^ (kg/m^2^). Participants were categorized as having obesity, with a BMI ≥ 30, or as non-obese, with a BMI < 30, based on established BMI thresholds. The categorization was in line with the World Health Organization’s international standards for defining obesity [46]. The selected SNP *MC4R* rs17782313 chosen for this study analysis was previously identified to have a significant association with obesity and was previously studied in regard to its role in eating behaviors and metabolic pathways [44,47,48]. The selection criteria for the SNP included a minor allele frequency (MAF) greater than 0.01 and validation in at least two genome-wide association study (GWAS) populations [35,36], as well as inclusion in the verified catalog of GWASs published [49]. The rigorous selection criteria ensured the reliability and validity of the genetic data used in the study. The Hardy–Weinberg equilibrium was assessed for this SNP via a chi-square test with 1 degree of freedom and was found to be in Hardy–Weinberg equilibrium.

### 2.3. Eating Behaviors Variables

Eating behaviors were assessed using an online validated self-reported questionnaire. The questionnaire was designed to capture a comprehensive range of eating habits and preferences, including frequency and type of meals, to better understand the behavioral aspects contributing to obesity. The questionnaire responses utilized a Likert scale to assess eating behavior statements, allowing participants to self-assess their eating habits across a range of behaviors. The items in the questionnaire were designed to probe various aspects of eating behaviors, including but not limited to cravings for sugary foods, tendencies toward emotional eating, instances of eating beyond satiety, frequency of consuming junk food at least once a week, rapid eating patterns, late-night eating habits, avoidance of eating at a designated dining table, eating in the absence of hunger, eating while in a standing position, and eating while engaged in distractions such as using a phone, watching television, or reading. For the analysis, we dichotomized each variable ranging from rarely to always.

### 2.4. Statistical Analysis

A preliminary power analysis was executed with the G*Power 3.1.9.7 software to determine the sample size for the study. Results indicated that a sample comprising 80 participants divided into two groups would be adequate to detect a relationship between SNPs and obesity, with a statistical significance level set at 0.05, an odds ratio (OR) of 2, and a power of 0.80. Descriptive statistics were presented as percentages for categorical variables, while continuous variables were displayed as mean ± standard deviation (SD). Differences between the two groups for continuous variables were tested using an independent sample *t*-test when data were normally distributed and a Mann–Whitney U test for non-normally distributed data. A Chi-squared test was applied to test for differences in categorical variables between the two groups. Logistic regression models adjusting for age, sex, type 1 diabetes mellitus (T1DM), and type 2 diabetes mellitus (T2DM) were performed to assess the association between *MC4R* rs17782313 with obesity risk. A logistic regression model was constructed to examine the interactions between the *MC4R* rs17782313 risk allele homozygous genotype in females and various eating behaviors in relation to obesity. This model was particularly focused on the female cohort due to the observed sex-specific associations. The model incorporated the dichotomized eating behavior variables and controlled for potential confounders: age, T1DM, and T2DM. ORs and 95% CI were calculated to quantify the strength and direction of these interactions. The statistical significance was set at an α level of 0.05, and any *p*-value below this threshold was considered to indicate a statistically significant difference between the groups under investigation. Analysis was performed using SPSS 29.0 and R software 4.3.1.

## 3. Results

### 3.1. Participants

The general characteristics of the study participants are described in Table 1. The mean age of the study cohort was 56.47 ± 14.48 years. Among the total cohort (*n* = 5785), the majority were female, accounting for 69.5% of the sample. This sex distribution was noteworthy and guided the focus of subsequent sex-specific analyses. Within the group with obesity, 67.2% were female, while in the non-obese group, 72.4% were female (*p* < 0.001). The mean weight of 98.34 and mean BMI of 35.29 were significantly higher in the group with obesity compared to 73.61 and 26.44 in the non-obese group, respectively (*p* < 0.001). The T2DM prevalence of 9% within the obesity group was significantly higher than the 6.3% in the non-obese group and thus was adjusted for in all analyses. T1DM did not differ significantly between the groups (BMI ≥ 30 and BMI < 30).

### 3.2. MC4R rs17782313 and Obesity Risk

As shown in Table 2, genotype frequencies of *MC4R* rs17782313 SNP were 57.83% for the reference homozygote TT, 35.49% for the heterozygote TC, and 6.68% for the altered homozygote CC among individuals with obesity. In the non-obese group, genotype frequencies were 59.1% for the reference homozygote TT, 35.76% for the heterozygote TC, and 5.13% for altered homozygote CC. Accepted genetic models were tested to determine the association of the *MC4R* rs17782313 SNP with obesity risk. Both the recessive and additive models showed a significant association with BMI (OR = 1.38 95% CI 1.1–1.72, *p* = 0.005 and OR = 1.1, 95% CI: 1.01–1.2, *p* = 0.03, respectively). Homozygous carriers of the risk allele exhibited a mean BMI of 31.96 ± 6.2, which was significantly higher than the wild type and one allele carriers, who had a mean BMI of 29.45 ± 6.49 (*p* = 0.02). Interestingly, after further stratification by sex, the obesity risk remained significant only among females in the recessive model (OR = 1.41, 95% CI: 1.09–1.82, *p* = 0.01), with an average of 0.85-BMI increment compared to wild type and one risk allele carriers. Under the dominant model, no significant association was observed (*p* = 0.16, OR = 1.08, 95% CI: 0.97–1.2). This sex-specific finding led us to focus our subsequent analyses primarily on the female cohort where the association was robust and statistically significant.

### 3.3. Interactions of MC4R rs17782313 with Eating Behaviors on Obesity Risk

The *MC4R* rs17782313 homozygous genotype significantly interacted with eating behaviors to enhance the risk of obesity. Specifically, these eating behaviors included sweets desire (1.47, 95% CI 1.05–2.07, *p* = 0.03), emotional eating (OR = 3.32, 95% CI 2.08–5.3, *p* < 0.001), eating to over fullness (OR = 2.32, 95% CI 1.39–3.87, *p* = 0.001), consuming junk food ≥1/week (OR = 3, 95% CI 1.36–6.67, *p* = 0.007), fast eating (OR = 1.64, 95% CI 1.06–2.54, *p* = 0.027), late-night eating (OR = 2.35, 95% CI 1.44–3.82, *p* < 0.001), non-tableside eating (OR = 1.72, 95% CI 1.05–2.8, *p* = 0.03), non-hunger-driven eating (OR = 1.76, 95% CI 1.19–2.6, *p* = 0.005), eating while standing (OR = 2.09, 95% CI 1–4.4, *p* = 0.05), and distracted eating (OR = 1.68, 95% CI 1.2–2.36, *p* = 0.003) (Table 3).

## 4. Discussion

Our study results demonstrate that *MC4R* rs17782313 homozygous carriers are associated with a significantly higher risk of obesity in Israeli females (OR = 1.38, 95% CI 1.1–1.72, *p* = 0.005). The association of *MC4R* rs17782313 with an elevated risk of obesity or other related measures is not consistent across different populations, as the causal factors of genetic variants vary across populations. In accordance with our study, results from a meta-analysis showed a significant association of *MC4R* rs17782313 with an elevated risk of obesity in Caucasians following the recessive model (OR = 1.52 95% CI 1.13–2.03, *p* = 0.005) [20]. In contrast, the controversial effect of *MC4R* rs17782313 on BMI was found in the Indian population. In the North Indian population, the elevated risk of obesity was elevated only for homozygous risk allele carriers compared to wild type (OR = 1.7, 95% CI 1–2.8, *p* = 0.02) [50]. Whereas in the Mizo tribe from the North-East Indian population, carrying at least one risk allele had a reduced risk for elevated BMI (OR = 0.39, 95% CI 0.2–0.76, *p* = 0.006). This controversy can be explained by a genetic predisposition owed to different genetic ancestry alongside lifestyle factors. This could be also reflected by the wide range of obesity frequency in the Indian population [51,52]. The association of *MC4R* rs17782313 carriers with elevated obesity risk follows different inheritance models in different studies’ findings. In a study of the Brazilian population, only the heterozygous showed a significantly elevated obesity risk [53]. Studies in other populations’ cohorts have found an elevated obesity risk following the dominant model including Manoan (OR = 1.43 95% CI 1.07–2.06, *p* = 0.02) [54], Arabs from the United Arab Emirates (OR = 1.35 95% CI 0.99–1.85, *p* = 0.054) [55], and Sri-Lankans (OR = 2.57, 95% CI 1.11–2.22, *p* = 0.01) [43].

Several studies have found that *MC4R* rs17782313 is sex-specific [56,57]. Thus, we further stratify by sex. We found that carrying the *MC4R* rs17782313 is associated with obesity risk only in females. Similar results were shown in other populations. A case-control study, which included 336 adult Pakistani males and 270 females, found that only female’s carriers of the rs17782313 *MC4R* genotype were at a significant 2.43- and 1.55-fold (95% CI: 1.19–4.96, *p* = 0.015, and 95% CI: 1.1–2.18, *p* = 0.01) risk of being overweight and having obesity, respectively [57]. Another study in Brazil showed a significant (*p* = 0.038) increased risk for obesity of the *MC4R* rs17782313 carriers only in females [53]. Additionally, a study by Thea Bjørnland et al. found that the effects of obesity-promoting genes like *MC4R* and their interactions with lifestyle factors are age- and sex-related [58]. Findings on an elevated risk of obesity among females compared to males can be explained by the findings of Horstmann et al., who demonstrated an increased ‘emotional eating’ score of the *MC4R* rs17782313’s variant risk allele carriers and suggested that the *MC4R* rs17782313 effect on eating behavior is mediated by central mechanisms that are sex-specific [59]. Along these lines, *MC4R* rs17782313 variant carriers were also associated with higher intakes of total energy among Caucasian females [60]. While these studies provide some evidence for sex-specific effects, more research is needed to establish a definitive link between *MC4R* rs17782313 and sex-specific obesity risk. The identification of *MC4R* rs17782313 as a significant risk factor for obesity, particularly among females, could pave the way for more targeted interventions. The lack of a significant association in males (*p* > 0.05) suggests that the *MC4R* rs17782313 SNP may not be a major determinant of obesity risk in men within our study population. This could be due to various factors, including, but not limited to, hormonal differences [61], lifestyle factors [62], or even the possibility of interactions with other genetic variants. It is also worth noting that obesity in men and women is associated with different neural mechanisms. While changes in somatosensory regions are more prevalent in men with obesity, reward regions of the brain show greater involvement in women [63]. These sex-specific neural responses, including those related to taste, could potentially influence the observed sex differences in the genetic predisposition to obesity.

Our detailed interaction analysis demonstrated significant obesity risk interactions between *MC4R* rs17782313 variant homozygotes and various and wide eating behaviors including sweets desire, emotional eating, overfullness, junk food consumption, fast eating, late-night eating, non-hunger-driven eating, eating while standing, and distracted eating. These interactions were a key finding of this study, shedding light on the complex interplay between genetic and behavioral factors in obesity, and are the first, to the best of our knowledge, that specify different and wide eating behaviors that compose a clearer eating behaviors category related to obesity risk genetic predisposition. These findings align with existing literature that has demonstrated the influence of this genetic variant on various specific eating issues. For instance, the *MC4R* rs17782313 variant has been demonstrated to be significantly associated with a higher prevalence of snacking as was shown in both French children with obesity (*p* = 0.01) and Swiss adults with obesity (*p* = 0.04), as well as in Finnish adolescents (*p* = 0.04). Furthermore, French adults’ carriers of the *MC4R* rs17782313 variant with familial obesity demonstrated significantly higher hunger scores (*p* = 0.02). Similarly, French children with obesity who are also carriers of this variant demonstrated a significantly higher prevalence of eating large amounts of food (*p* = 0.04) [39]. Furthermore, our findings are consistent with a study conducted on an Iranian cohort, which also found significant interactions between emotional eating and the *MC4R* rs17782313 CC genotype in terms of BMI, further substantiating the role of emotional eating in the complex relationship between this genetic variant and obesity [47].

It is important to acknowledge the inherent limitations of this study. Specifically, the cross-sectional nature of this study restricts our ability to establish causal relationships between the variables examined. This study’s advantages include the large, population-representative cohort, which enabled us to analyze *MC4R* polymorphism and the eating behaviors interactions effect associated with obesity risk. Additionally, our findings are the first, to the best of our knowledge, that specify different and wide eating behaviors that compose a clearer eating behaviors category related to the genetic predisposition of a risk of obesity. This adds a new dimension to our understanding of how genetic factors and eating behaviors interact to influence obesity risk and paves the way for further research that could potentially lead to targeted interventions based on specific eating behaviors influenced by genetic markers. Furthermore, as the causal factors of genetic variants vary across populations, our findings shed light on the Israeli population, which, to the best of our knowledge, was not investigated regarding the effect of *MC4R* rs17782313 and on obesity risk. Given that genetic factors can manifest differently across diverse populations, our study lays the groundwork for the nutritional tailored unique genetic makeup of different populations.

## Figures and Tables

**Table 1:** Descriptive characteristics of study participants.

	All Population *n* = 5785	With Obesity (BMI ≥ 30) *n* = 3157	Non-Obese (BMI < 30) *n* = 2628	*p*-Value
Sex (women, %)	4023 (69.5%)	2120 (67.2%)	1903 (72.4%)	<0.001
Age (mean ± SD)	56.47 ± 14.48	56.61 ± 14.66	56.31 ± 14.26	0.22
Weight (mean ± SD)	87.1 ± 19.4	98.34 ± 17.7	73.61 ± 10.93	<0.001
Height (mean ± SD)	166.64 ± 8.85	166.69 ± 9.12	166.58 ± 8.52	0.86
BMI (mean ± SD)	31.27 ± 6.04	35.29 ± 5.05	26.44 ± 2.6	<0.001
T1DM (n, %)	100 (1.73%)	62 (1.96%)	38 (1.45%)	0.27
T2DM (n, %)	449 (7.76%)	284 (9%)	165 (6.3%)	<0.001

**Table 2:** *MC4R* rs17782313 (T>C) genotype frequencies and obesity risk among the total sample and stratified by sex.

	Genotype Frequency (%)				*p*-Value OR ± 95%(CI)		
Sample	Genotype	Overall Population	With Obesity (BMI ≥ 30)	Non-Obese (BMI < 30)	Dominant Model	Recessive Model	Additive Model
All sample *	TT	3375 (58.3%)	1818 (57.6%)	1557 (59.2%)	0.16 1.08 (0.97–1.2)	0.005 1.38 (1.1–1.72)	0.03 1.1 (1.01–1.2)
	TC	2062 (35.6%)	1125 (35.6%)	937 (35.6%)			
	CC	348 (6%)	214 (6.8%)	134 (5.1%)			
Females (*n* = 4023)	TT	2328 (35.6%)	1211 (57.1%)	1117 (58.7%)	0.28 1.07 (0.95–1.22)	0.01 1.41 (1.09–1.82)	0.06 1.62 (1.24–2.11)
	TC	1434 (35.6%)	751 (35.4%)	683 (35.9%)			
	CC	261 (6.5%)	158 (7.4%)	103 (5.4%)			
Males (*n* = 1762)	TT	1047 (59.4%)	607 (58.5%)	440 (60.6%)	0.34 1.1 (0.9–1.34)	0.28 1.28 (0.82–1.01)	0.23 1.11 (0.94–1.23)
	TC	628 (35.6%)	374 (36.1%)	254 (35.0%)			
	CC	87 (4.9%)	56 (5.4%)	31 (4.3%)			

Adjusted for age, T1DM, and T2DM; * adjusted for age, sex, T1DM, and T2DM.

**Table 3:** Eating behavior prevalence among *MC4R* rs17782313 (CC) females’ carriers stratified by BMI and the interaction effect on obesity.

Eating Behavior	BMI ≥ 30	BMI < 30	β	OR ± 95% CI	*p*-Value
Sweets desire	91 (57.6%)	56 (54.4%)	0.387	1.47 (1.05–2.07)	0.03
Emotional eater	83 (52.5%)	23 (22.3%)	1.2	3.32 (2.08–5.3)	<0.001
Overfullness feeling	54 (34.2%)	21 (20.4%)	0.84	2.32 (1.39–3.87)	0.001
Junk food ≥ 1/week	26 (16.5%)	8 (7.8%)	1.1	3 (1.36–6.67)	0.007
Fast eater	57 (36.1%)	32 (31.1%)	0.495	1.64 (1.06–2.54)	0.027
Late night eater	60 (38%)	23 (22.3%)	0.853	2.35 (1.44–3.82)	<0.001
Non-tableside eater	48 (65.8%)	25 (34.2%)	0.541	1.72 (1.05–2.8)	0.03
Non-hunger-driven eater	75 (47.5%)	39 (37.9%)	0.565	1.76 (1.19–2.6)	0.005
Eat while standing	23 (14.6%)	10 (9.7%)	0.736	2.09 (1–4.4)	0.05
Distracted eater	98 (62.0%)	54 (52.4%)	0.519	1.68 (1.2–2.36)	0.003

Adjusted for age, T1DM, and T2DM.

## Data Availability

Data sharing is not applicable to this article.
